# Artificial intelligence-driven advancements in agricultural biotechnology

**DOI:** 10.1016/j.jgeb.2026.100749

**Published:** 2026-06-26

**Authors:** Zubaer Hossen, Md. Naim Uddin Forhad, Md. Rifat Bin Ayez, Shusmita Karmaker, Md Nur Islam, Md. Sarowar Hossain, Md. Enamul Haque, Md. Nazmul Hasan, Md Mahmudul Islam

**Affiliations:** a Department of Biotechnology and Genetic Engineering, Gopalganj Science and Technology University, Gopalganj-8105, Bangladesh; b Department of Biochemistry and Molecular Biology, Gopalganj Science and Technology University, Gopalganj-8105, Bangladesh; c Department of Genetic Engineering and Biotechnology, Faculty of Health and Life Sciences, Daffodil International University, Dhaka 1216, Bangladesh; d Department of Pharmacy, Manarat International University, Dhaka 1341, Bangladesh; e Computational Biology Research laboratory, Department of Pharmacy, Faculty of Health and Life Sciences*,* Daffodil International University*,* Dhaka 1216*,* Bangladesh

**Keywords:** Artificial intelligence, Farming, Agriculture, Biotechnology, Ethics, Climate

## Abstract

The need for faster and more informative data processing for better decision-making is driving the adoption of artificial intelligence (AI) in the agricultural sector. Thanks to recent advancements in computer science and the increase in computational powers of modern computers, AI is not only augmenting traditional solutions, but also helping in developing novel solutions to existing challenging matters. AI-driven models have an exceptional ability to identify patterns and combine a diverse collection of data together and make inference. The increasing pressure on farmlands posed by the growing global population and climate change is lessening growth, yield, and productivity ultimately posing risk to food security worldwide. Incorporation of AI in agriculture has the potential to drive farming efficiency to new heights. This comprehensive review critically evaluates the evolution of AI in agricultural biotechnology from a theoretical concept to a global phenomenon. A comprehensive literature search was performed using major scientific databases, including PubMed, Web of Science, Embase, Scopus, Lens and the Cochrane Library. In this review, we empirically demonstrate the fields advancement toward more capable AI systems and discuss the current applications of AI across crop improvement and precision agriculture such as crop improvement and genetic engineering, genomic selection and plant breeding, pest and disease detection, precision agriculture and smart farming, soil health and nutrient management, climate resilient crop development, livestock biotechnology, challenges and ethical considerations in AI based agricultural biotechnology. Furthermore, this review addresses the exponential growth of commercial intellectual property in the field and contrast it with academic publication outputs. Finally, we critically assess the ethical challenges impeding equitable adoption of AI including data sovereignty and digital divide, while projecting future frontiers involving quantum computing. This review will help build sustainable agricultural systems capable of adapting to climate change, contribute to the development of climate-resilient and high-yielding crops, and address global food security challenges.

## Graphical abstract

**Figure:**
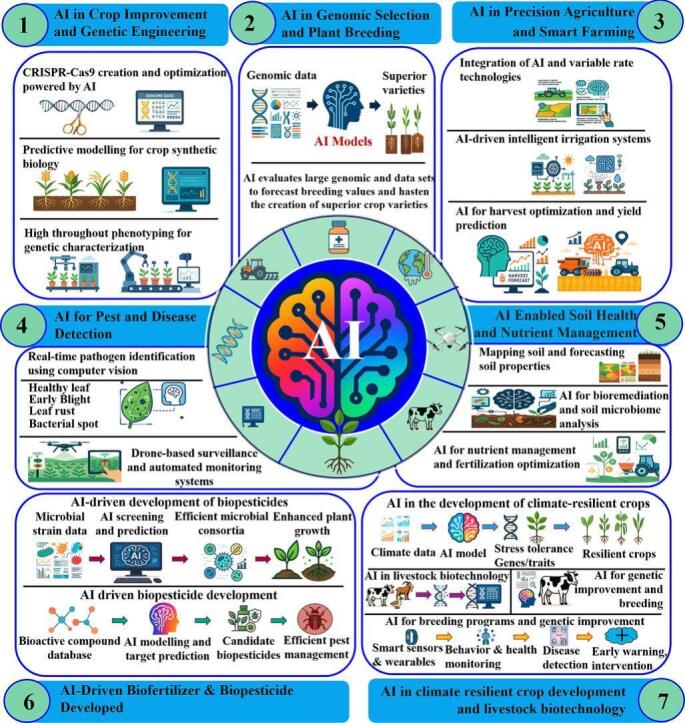

## 1 Introduction

Recent advancements in computer science have accelerated the development of artificial intelligence (AI), leading to rapid progress over the past few years. These advancements have initiated the incorporation of AI in agriculture and leading to a new phase of discovery and innovation. As such, AI is now offering data driven novel and scalable solutions to address critical challenges worldwide, including global food security [1].

AI powered technologies are increasingly being employed to optimize crop breeding programs, improve disease and pest detection and enhance the efficiency of resource utilization in agricultural systems [2]. AI powered platforms are forming the backbone of precision agriculture and smart farming systems to ensure higher yields and reduced environmental impact, practices crucial for feeding a growing global population amidst challenging climate conditions [3].

This review examines the core methodologies and computational architectures of today's AI systems and aims to provide a comprehensive overview of the AI enhanced advancements in agricultural biotechnology. This discussion begins with a definition of AI and an explanation of its fundamental operational principles. We subsequently analyze the contemporary implementations of AI within the agricultural sector followed by a critical assessment of the ethical barriers to widespread adoption of AI and try to shed light on the future directions of AI application in the said field. By drawing upon recently published literature and technological developments from 2024 to 2026, this article aims to serve as a valuable resource for scientists, policymakers and stakeholders in the industry seeking to navigate the rapidly evolving integration of AI in modern biotechnology.

## 2 Artificial intelligence

### 2.1 Definition of artificial intelligence

AI does not represent a single, unified technology; rather, it encompasses a broad range of computational methods and systems developed to emulate human cognitive capabilities [4]. In general terms, AI can be defined as the capability of computer systems to execute tasks that typically require human intelligence, including perception, logical reasoning, learning from past experience, and solving complex challenges [5].

#### 2.1.1 Historic development and evolution of AI

The historic progression of AI can broadly be divided into three phases

a. **Rule-Based AI (1950s–2000s):** Early AI operated on predefined logical structures and rules implemented through “if-then” statements. These were effective for repetitive tasks. However, they lacked the adaptability to manage variability inherent to many real world datasets 6, 7, 8, 9.
b. **Predictive AI (2000s–2020s):** With the emergence of machine learning (ML), the focus of AI research shifted from rule-based systems to a data driven approach. During this time, Algorithms were increasingly designed to analyze large datasets and detect patterns that could be used for predictive tasks such as forecasting crop yields 10, 11, 12, 13, 14.
c. **Generative and Agentic AI (2020-Present):** Now AI is largely characterized by Generative AI (GenAI) and Agentic AI systems. These AI systems can autonomously generate novel information and execute complex, multi-step tasks. Such capabilities are largely enabled by large scale pretrained models trained on extensive datasets [15].

**Fig. 1:**
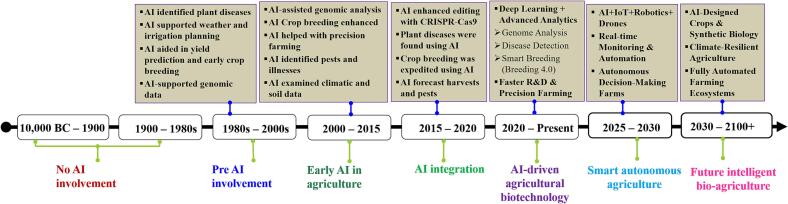
Timeline of AI integration and agricultural biotechnology development (10,000 BCE–2100+).

To provide a comprehensive visual representation of this historical progression, Fig. 1 delineates the major milestones in the integration of AI within the agricultural sector. This timeline extends from the pre-AI era to the anticipated future of intelligent bio-agriculture, showcasing the shift from rule-based systems to advanced neural architectures and autonomous farming ecosystems.

#### 2.1.2 Narrow AI versus artificial general intelligence (AGI)

Majority of AI currently in use are “narrow” or domain specific. They are optimized for specific, well defined tasks and operate within a limited scope of functionality [16]. AGI in contrast represents a theoretical class of AI system capable of understanding, learning, and applying knowledge across a variety of domains with cognitive capacities matching or superior to human intellect 17, 18.

#### 2.1.3 Quantitative insight into the shifting paradigm

**Fig. 2:**
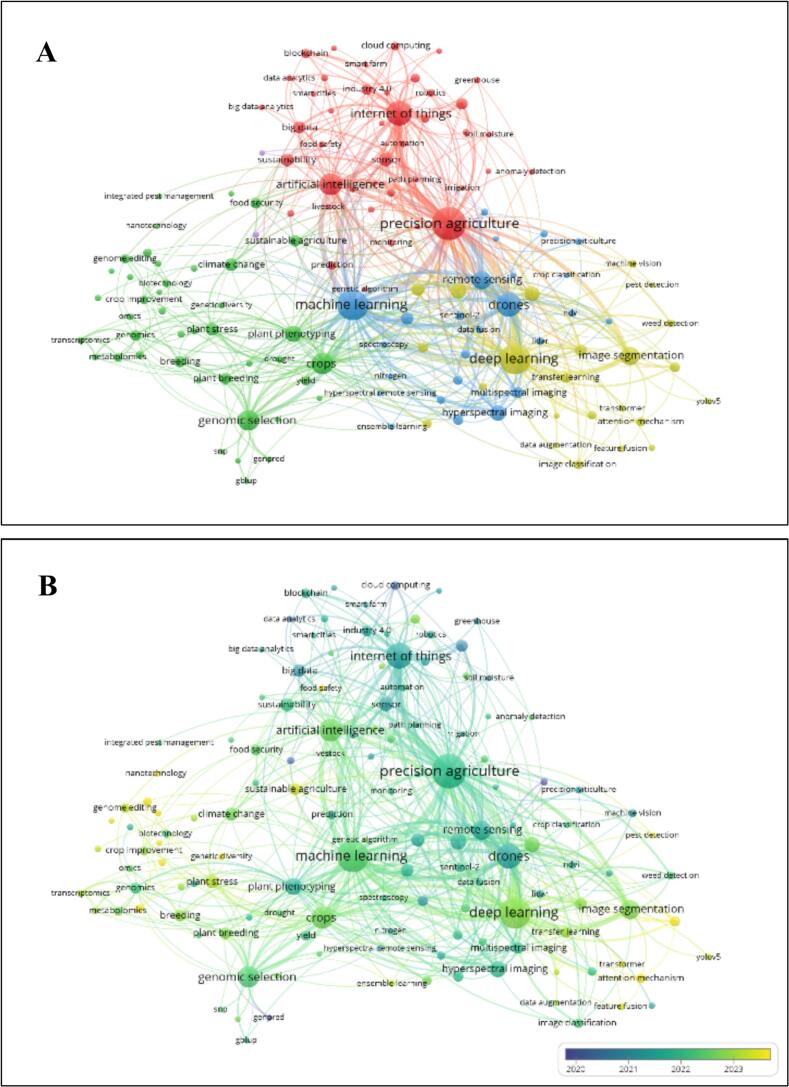
(A) Bibliometric co-occurrence network of 13,168 scholarly articles highlighting thematic clustering in AI-driven agricultural biotechnology. (B) Temporal overlay visualization demonstrating the paradigm shift from foundational machine learning to advanced neural architectures.

Note: Node color gradient represents the volume-weighted average publication year for each thematic cluster; while data extraction spans 2015–2025, the mathematical averaging anchors the maximum visual gradient at the median distribution point of the newest architectures.

To empirically map the evolution and integration of AI in the agricultural sector, a bibliometric network analysis was conducted using a dataset of 13,168 peer-reviewed journal articles and reviews published between January 2015 and December 2025. Using the keywords: (“artificial intelligence” OR “machine learning” OR “deep learning” OR “computer vision” OR “explainable AI”) AND (“agricultural biotechnology” OR “crop improvement” OR “genomic selection” OR “precision agriculture” OR “smart farming” OR “biopesticide”). A systematic search was performed in “scholarly works” in Lens (https://www.lens.org). Flag was set to “Has abstracts,” and Publication Type was set to “journal article” and “review.” This yielded the aforementioned number of results which were then subsequently analyzed in VOSviewer (https://www.vosviewer.com) version 1.6.20. As visualized in the keyword co-occurrence network (Fig. 2A), the literature has consolidated into several interconnected macro-clusters: precision agriculture, artificial intelligence and IoT (red), machine learning and imaging (blue), and genomic selection (green). This demonstrates that AI is no longer an isolated technology, but a system deeply integrated across multiple biotechnological applications. Further, a temporal overlay of this network (Fig. 2B) explicitly maps the chronological progression of this paradigm shift. It is important to note that while the extracted dataset encompasses literature until December 2025, the network's temporal gradient plots the *volume-weighted average* publication year (the chronological center of mass) for each node. Foundational concepts such as “machine learning,” “remote sensing,” and “internet of things” demonstrate a high-volume center of mass circa 2023. Emerging nodes such as “deep learning,” “genome editing,” “transformer,” and “nanotechnology” represent the current cutting edge, exhibiting a center of mass extending into late 2023 and beyond. This finding confirms that the sheer volume of recent publications is heavily concentrated in complex autonomous systems, marking an industry-wide transition away from basic atoms.

### 2.2 Core methodologies and computational architectures of AI

The effectiveness of AI arises from a set of methodologies and computational architectural frameworks. These frameworks facilitate the processing of high dimensional, multimodal datasets including environmental and phenotypic data to uncover previously unknown relationships and the automation of workflows 19, 20, 21, 22, 23.

#### 2.2.1 Fundamentals of machine learning

Machine Learning encompasses the foundational algorithmic models that empower computers to recognize patterns and draw inference. In general, they are classified by how they work with training datasets [24].

• **Supervised Learning:** Relies on labeled datasets in which each input is paired with a known output. In this framework, the model is trained by providing it both the input variables and their corresponding target values, allowing the model to iteratively minimize the error gradient between its predictive output and the actual data [25].
• **Unsupervised Learning:** Operating on unannotated, raw data, unsupervised models autonomously map the latent structure of a dataset. These models analyze the intrinsic properties of the data to identify natural groupings, associations or latent features [26]. Such approaches are valuable when manual data annotation is impractical or impossible. For example, this technique is widely used to detect heterogeneous soil zones for variable rate fertilization in precision agriculture 27, 28.
• **Reinforcement Learning (RL):** RL operates in an interactive learning paradigm in which an “agent” learns to make sequential decisions within a dynamic environment [29]. The system improves its performance through a process of trial and error, receiving positive feedback (rewards) for desirable actions and negative feedback (penalties) for unfavorable ones. Over time, the agent develops strategies that maximize cumulative rewards and optimize decision making [30].

#### 2.2.2 Deep learning architectures

Deep learning (DL), a branch of machine learning, employs artificial neural networks composed of multiple hidden layers. These layered architectures enable models to automatically learn complex, non-linear representations directly from raw and often unstructured data such as images, audio or sensor outputs without the need for manual feature engineering.

• **Convolutional Neural Networks (CNNs):** CNN's are designed for processing data with grid like structures, such as image based data. They use convolutional filters to extract hierarchical spatial features from raw input data [31]. Through the stacking of multiple convolutional network layers, the neural network can recognize high level, complex structures within images [32].
• **Recurrent Neural Networks (RNNs):** RNN's are optimized for sequential data processing. They incorporate internal memory states that retain information from previous inputs, enabling the network to account for temporal dependencies within a sequence [33]. As each step in the sequence is influenced by preceding inputs, RNN's are well suited for analyzing time dependent data, such as environmental monitoring data [34].
• **Transformers:** They bypass the sequential processing of RNNs via a self-attention mechanism. This architecture processes sequences in parallel, and assigns varying levels of importance to different elements of the input data, capturing long-range relationships more effectively [35]. Owing to their efficiency and scalability, transformer based models have become the dominant architecture in many large scale DL systems [36].

#### 2.2.3 Natural language processing (NLP) and large language models (LLMs)

NLP is an interdisciplinary field that integrates principles from linguistics, computer science and machine learning to enable computers to interpret, analyze and generate human language [37]. NLP typically transform raw, unstructured textual information into structured, machine interpretable representations through a series of computational steps. These steps commonly include tokenization, which divides text into smaller linguistic units; embedding, where tokens are converted into numerical vector representations; and semantic analysis, which determines contextual meaning and relationship within the text [38]. NLP, through these techniques, enable the extraction of automated large scale data from unstructured source [37].

#### 2.2.4 Graph neural networks and knowledge graphs

Unlike conventional neural networks that process grid based or sequential data, GNN's are designed to analyze data structured as graphs composed of nodes (entities) and edges (relationships) 39, 40. GNNs work a process called message passing where each node iteratively gathers and aggregates information from its neighboring nodes to update its internal representation. Through this process, the model can effectively capture the local structural and functional context surrounding a given entity, such as chemical interactions or biological relationships [41]. GNNS's are useful in applications where the spatial organization among components is more informative than the isolated properties of individual elements [42].

A knowledge graph is a structured data framework that represents information as a network of interconnected entities and their relationships. Within a KG, knowledge is stored in the form of triples, consisting of a subject, a predicate and an object [43]. They provide a curated domain specific information while GNNs can function as a reasoning layer capable of inferring previously unknown relationships [44].

### 2.3 Working principles of AI relevant to biotechnology

#### 2.3.1 Data representation and feature extraction

Raw biological data, whether it is a drone image of a farm, or image of a pest, is inherently complex and noisy. Before any meaningful analysis, this raw data must be transformed into something easily interpretable by computers. This transformational process, referred to as “data representation” involves converting inputs into high dimensional numerical structures such as vectors, embeddings or tensors that can be further processes by ML algorithms [45]. Rather than relying on manually defined descriptors, DL systems ae capable of automatically identifying and extracting the most informative patterns within the data 46, 47. For example, in agricultural biotechnology, algorithms such as vision transformers and CNNs process high resolution images to automatically extract phenotypic traits. These models can detect the precise pixel variations associated with subtle leaf wilt under early water stress long before the symptoms are visible to the human eye [48].

#### 2.3.2 Predictive classification via supervised learning

Upon extraction of relevant features, the AI utilize supervised learning to associate these inputs to specific outcomes. The model is exposed to large collections of labeled samples during training allowing it to learn the relationship between input features and corresponding outputs. Through repeated iterations, the neural network refines its internal parameters, namely the weights and biases using optimization procedures such as “backpropagation” to reduce prediction error. This allows the models to progressively learn highly complex, non-linear mapping functions that enable accurate predictions from previously unseen data [49]. In agriculture, CNNs trained on visual datasets of infected plant tissues are deployed to classify and diagnose crop pathogens such as tomato late blight or bacterial canker [50].

#### 2.3.3 Optimization and the design-build-test-learn cycle

**Fig. 3:**
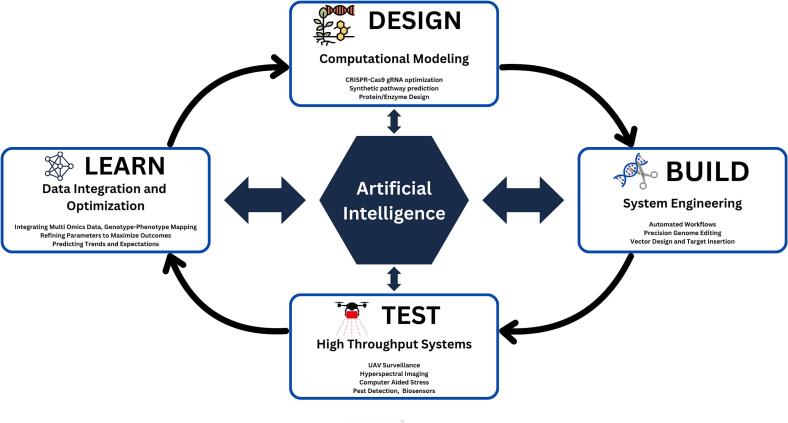
Conceptual architecture of the AI enabled Agri-Tech DBTL cycle with AI as the engine driving in silico prediction, automated system engineering, high throughput data analysis systems and continuous optimization.

The Design-Build-Test-Learn (DBTL) cycle is the foundational iterative framework for developing and optimizing biological systems [51]. Traditional DBTL cycles are severely bottlenecked by manual laboratory processes. However, as illustrated in Fig. 3, the integration of AI accelerates every phase of this cycle. By predicting the outcomes of experiments in silico during the Design phase, guiding automated synthesis in the Build phase, processing high-throughput data during the Test phase, and mapping multi-omics data in the Learn phase, AI effectively closes the loop. This continuous data ingestion allows researchers to bypass the traditional trial-and-error approach, focusing valuable resources only on the most promising candidates, enabling rapid innovation [52].

### 2.4 Data-driven approach in AI

#### 2.4.1 Big data in Agri-tech

Modern agriculture increasingly relies on large and diverse datasets to enhance productivity and optimize land use efficiency [53]. Agricultural data ecosystems integrate multiple sources of information such as spatial datasets derived from satellite based multispectral imagery, temporal environmental measurements from localized meteorological stations and biological datasets generated through soil microbiome sequencing and high throughput plant phenotyping platforms [54]. As such, the primary computational challenge has shifted from data collection to the integration and interoperability of these heterogeneous data streams. Developing unified frameworks capable of interpreting sch large, disparate datasets is essential for enabling AI systems to generate useful insights in agricultural settings [55].

#### 2.4.2 Data processing and feature engineering

Raw agricultural data is characterized by noise, sparsity, and biases like batch effects that arise during data collection or processing 56, 57. Rigorous preprocessing is necessary before such data can be used for AI training. These preprocesses usually include normalization, imputation of missing values and removal of technical artifacts that may distort downstream analyses [58]. After preprocessing, to convert aw data into mathematical representations suitable for machine learning algorithms, feature engineering is employed 59, 60. Traditionally this is mostly a manual process heavily dependent on domain expertise to identify informative variables.

However, recent developments in DL have shifted toward automated feature extraction. In agricultural computer vision, convolutional layers autonomously engineer features to detect subtle variations in leaf morphology or canopy temperature, bypassing the need for manual phenotypic annotation 61, 62, 63.

#### 2.4.3 Model training, validation and evaluation

A data driven AI workflow involves not only training algorithms on large datasets but also rigorously validating their predictive performance. In agro-tech, analysts frequently encounter the curse of dimensionality, a situation in which the number of features greatly exceeds the number of available samples [64]. This imbalance substantially increase the risk of overfitting where a model learns the patterns of the training data too closely and consequently performs poorly when applied to new, previously unseen data [64]. To address this, model development typically involves strict data partitioning strategies and validation techniques such as k-fold-cross-validation, which iteratively evaluate the model on previously unseen subsets of the dataset [65].

In crop disease detection, metrics such as the Area Under the Receiver Operating Characteristic Curve (AUC-ROC), Precision, Recall and the F1-score are critical, as they account for imbalanced datasets where a disease state might be rare compared to healthy controls [66]. Moreover, modern regulatory standards increasingly demand external validation, testing the trained model on entirely independent geographic or demographic cohorts, to ensure the AI performs reliably across varied agricultural climates [67].

## 3 Artificial intelligence in agricultural biotechnology

### 3.1 AI in crop improvement and genetic engineering

#### 3.1.1 AI-driven CRISPR-Cas9 design and optimization

The emergence of AI used in combination with CRISPR-Cas9 tools upgraded the preciseness and efficiency of developing more productive crop lines [68]. AI's ability to handle data, spot patterns, and make predictions has an important role in optimizing every part of the process from finding targets, crafting guide RNAs, even “guessing” where edits might go wrong [69]. Instead of long, manual lab checks that used to be standard, new AI methods quickly point to the best gRNA options while also flagging places where unintended changes could happen, often with high precision [70].

**Fig. 4:**
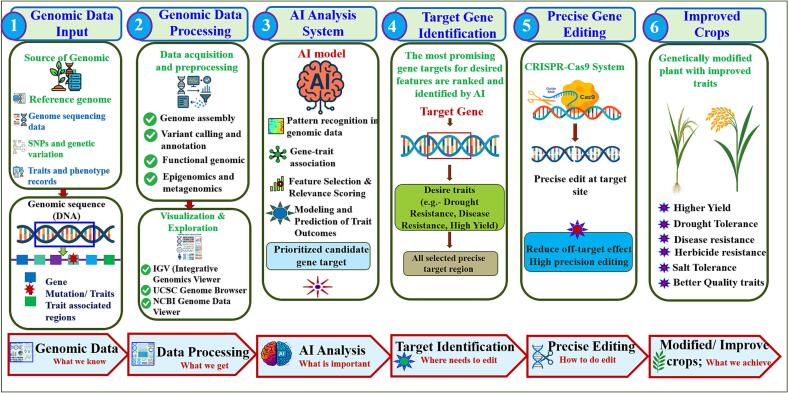
Development of superior crop varieties using AI-driven genomic data and CRISPR-Cas9 technology. Using AI to analyze large genomic data sets to identify key genes and desirable agronomic traits such as yield, stress tolerance, and disease resistance. Selected gene targets are then validated and edited using CRISPR-Cas9 to introduce specific changes in the plant genome. The edited plants are phenotypically screened and evaluated at high throughput to select the superior crop lines with improved productivity, resilience, and nutritional quality.

In plant gene editing, machine learning tools are now developed to guess how well a gRNA will work, helping cut down on mistakes, making plant gene editing safer and less error prone [71]. Massive genetic data is fed into these AI systems including details like DNA patterns, how chromatin is opened or closed, chemical tags on genes. From all that, the AI pick out gRNA shapes that work best for disabling specific genes or adding new sequences [72]. AI also helps fine-tune Cas9 versions along with different CRISPR pieces. Scientists now use AI to create new Cas9 enzymes that are more precise and can reach more genetic locations [73]. The systematic integration of AI in the genomic editing pipeline is illustrated in Fig. 4. This workflow demonstrates the transition from raw genomic data acquisition to the identification of target genes and the subsequent application of high-precision CRISPR-Cas9 editing to achieve desired crop traits.

AI tools examine protein shape, predicts how changing amino acids will affect the shape, then steer the process of developing Cas9 [74]. Tools like Protein MPNN quickly identify potential amino acid lists for building proteins, saving time and allowing flexibility when crafting better Cas9 types. Because AI can handle huge, tangled biological datasets, picking the right CRISPR tools now happens in a flash, speeding up how new crops are designed that has higher yield, resist disease, or carry more nutrients [75]. Through the quick generation of climate resilient and high yielding crop varieties AI is transforming plant breeding by helping creating new opportunities to address global food security challenges [76].

#### 3.1.2 Predictive modeling for synthetic biology in crops

AI-driven predictive modeling goes beyond the old-style trial and error methods by using computational tools to predict how engineered genetic circuits and metabolic pathways will behave inside plants 74, 77. ML and DL algorithms allow researchers to analyze large genomic, proteomic, and metabolic datasets and build models that can predict phenotypic outcomes [78]. For example, AI can model how a new plant variety will perform in certain environments even before it is fully grown. This helps speed up the breeding process and improves the chances of attaining better crops [79]. This predictive ability is crucial for designing crops with better traits like higher yield, improved nutrition, and stronger resistance to environmental stress [80].

One use of predictive modeling is optimizing gene expression and metabolic activity in engineered plants. AI can identify the best genetic setups and regulatory elements needed to drive important biochemical processors, such as boosting photosynthesis or improving nutrient uptake [81]. Additionally, AI enabled predictive models are used to design new proteins and enzyme with specific functions, which can be later integrated into synthetic biological pathways to give crops new traits [76].

The combination of AI and synthetic biology also makes it easier to develop automated bioengineering workflows, where AI can manage each step of the DBTL cycle for engineered microbes or plants with minimal human supervision [77]. This repetitious process driven by predictive AI models allows synthetic biological systems to be prototyped and improved quickly.

#### 3.1.3 High throughput phenotyping for genetic characterization

AI assisted high throughput phenotyping (HTP) is bridging the gap between genotype and phenotype to determine genetic traits ^[82]^. Traditional phenotyping methods demand manual work by humans, which is time-consuming, expensive, and limited in their ability to capture dynamic plant traits across different environments. HTP systems, on the other hand, use advanced sensing technologies, robotics and imaging systems to quickly and non-invasively collect large amount of data on plant growth, development and stress responses ^[83]^. This flood of data including parameters like leaf area, plant height, biomass, and physiological indices, requires advanced analytical tools, which sophisticated AI algorithms can provide ^[84]^**.** Machine learning and DL algorithms are especially skilled at processing and interpreting these complex datasets, extracting meaningful insights that would otherwise be impossible to detect ^[85]^. AI powered HTP lets breeders spot important genetic traits quickly and accurately speeding up the development of better crops. Computer vision, a branch of AI, analyzes images from HTP platforms, automatically measuring complex traits and detecting subtle differences that show genetic variation or stress responses ^[86]^. These models can predict how plants perform in different environments, helping identify genes linked to drought tolerance disease resistance and nutrient efficiency ^[87]^.

### 3.2 AI in genomic selection and plant breeding

Machine learning models have become indispensable tools for genomic prediction in plant breeding, reshaping how improved crop varieties are selected ^[88]^. The goal of genomic prediction is to predict the genetic merit of individuals using their genomic information thereby accelerating breeding cycles and increasing the accuracy of selection ^[89]^. Currently available breeding methods rely on phenotypic selection, which is time consuming and heavily influenced by environmental factors such as water availability, salinity and soil pH. ML models can integrate large and complex genomic, phenotypic and environmental datasets to build predictive models that accurately estimate breeding values ^[90]^. DL architectures such as CNNs and RNNs have shown strong performance in capturing non-linear relationships and complex interaction within genomic data often outperforming traditional statistical approaches ^[90]^. These models can efficiently handle high dimensional genomic datasets, identify key genetic markers associated with desirable traits, and predict the performance of non-phenotyped individual or new crosses ^[91]^. Machine learning has been applied across many areas of plant breeding from predicting yield and disease resistance to estimating how well a variety will perform in specific environment ^[92]^. For example, ML models trained on historical breeding data can forecast how new genotypes will behave under different conditions, giving breeders valuable guidance on which genetic line is worth advancing ^[93]^. MLs ability to combine multi omics datasets adds another layer of filter to genomic predictions by offering a more complete picture of the genetic basis of complex traits ^[94]^.

AI algorithms are accelerating the breeding cycle too, essentially becoming an important factor in developing new crops varieties faster ^[89]^. Traditional plant breeding is a slow and lengthy process often taking years or even decades to develop and release a new variety, as each generation go through repeated round of selection and evaluation 92, 95. AI powered genomic prediction models can forecast the performance of crop lines based on the genetic profiles, allowing breeders to identify promising candidates without relying on extensive multi-year field trials ^[93]^. This predictive capabilities shorten the breeding timeline significantly as only the most promising lines are advanced, reducing the time and resources spent on inferior genotypes ^[96]^. Moreover AI algorithms help to integrate rapid generation advancement (RGA) techniques, in which plants are grown under optimized condition that shorten their life cycle and paired with precise selection strategies ^[97]^. By combining RGA with AI powered phenotyping and genomic selection, breeders can produce multiple generation per year reducing the overall breeding timeline significantly ^[98]^. AI also plays a key role in optimizing experimental design and resource allocation within breeding programs, ensuring that effort are focused on maximizing genetic gain per unit of time ^[99]^. Its ability to analyze the complex interaction among genes, environments and management practices enable the development of climate resilient, high yielding crops at unprecedented speed ^[100]^.

### 3.3 AI for pest and disease detection

#### 3.3.1 Computer vision for real time pathogen identification

Current pathogen detection methods rely heavily on manual inspection, which is labor intensive, slow and prone to human error; factors that often contribute to significant crop losses ^[101]^. AI powered computer vision (CV) systems use advanced image processing capabilities to analyze visuals collected from drone, ground robots and even smartphones ^[102]^. CNNs can learn complex pattern and features associated with specific pathogens from large datasets of annotated images ^[103]^. This allows for fast and accurate disease classification, distinguishing between different types of infections and even abiotic stresses that may mimic disease symptoms ^[104]^. CV is especially important for pathogen detection in precision agriculture, as it allow farmers to take targeted action immediately. For instance, CV based systems can be deployed directly in the field to continuously monitor crop health and send instant alert the moment a potential disease outbreak is detected ^[105]^. This not only limits the spread of pathogens but also reduce the need for broad spectrum pesticide use, supporting more sustainable and environmentally friendly farming practices ^[106]^**.** In addition CV can be integrated with other AI tools such as predictive analytics to forecast disease progression and optimize treatment strategies ^[107]^.

#### 3.3.2 Automated monitoring systems and drone based surveillance

Automated monitoring systems represent a major leap in agriculture. Compared to manual scouting, automated monitoring systems offer an efficient, scalable, and highly precise way to assess crop health across large areas ^[108]^. Drones equipped with high resolution cameras and multispectral or hyperspectral sensor capture vast amount of data, including visual imagery, thermal signatures, and spectral reflectance. This information can reveal early symptoms of stress, disease, or pest infestation that are often difficult for humans to find out before the symptoms are widespread ^[109]^. Algorithms, particularly those based on CV and DL can accurately detect and classify plant diseases, distinguish between different pest species and even quantify the severity of an infestation ^[110]^. This real time data driven approach enables farmers to make informed decision and apply targeted interventions, reducing crop losses and optimizing resource use. The integration of AI with drone technology enables continuous and autonomous field monitoring, allowing issues to be detected early long before they manifests into serious losses ^[111]^. For example, AI powered drones can identify fungal infections, nutrient deficiencies, or insect damage at their initial stages, making it possible to apply treatments precisely and reduce overall pesticide usage ^[112]^. These system can also track the progression of disease populations over time, providing valuable data for predictive modeling and integrated pest management strategies ^[113]^.

### 3.4 AI in precision agriculture and smart farming

#### 3.4.1 Variable rate technology and AI integration

Variable Rate Technology (VRT), has been significantly enhanced by its integration with AI, enabling a more precise and efficient application of agricultural inputs ^[114]^. VRT allow farmers to apply resources such as fertilizer, water, pesticides, and seeds variably across a field, tailored to the specific needs of different zones rather than applying at a uniform rate across the entire area ^[115]^. This site specific management optimizes resources use, reduces waste and minimizes environmental impact. ^[116]^.

By precisely applying fertilizers based on AI analyzed soil nutrient levels, farmers can prevent over fertilizing in some areas and under-fertilizing in others, leading to healthier crops and reduced nutrient runoff ^[117]^. Similarly, AI powered VRT for irrigation can optimize water usage by delivering water only where and when it is most needed, conserving water ^[118]^. The technology also extends to variable rate seeding, where AI determines optimal seeding densities based on soil type, historical yield, and other environmental factors, maximizing productivity ^[119]^.

#### 3.4.2 Smart irrigation systems driven by AI insights

Smart irrigation systems help farmers use water more efficiently while supporting healthier and more productive crops ^[120]^. In traditional irrigation setups, water is applied evenly across an entire field, even though different areas may have very different moisture requirements. This often leads to wasted water and inconsistent crop growth ^[121]^. AI based irrigation systems change this by combining data from sensors with advanced analytics and ML models ^[122]^. They monitor conditions such as soil moisture, local weather patterns, evapotranspiration, and growth stages of crops. By bringing these data sources together, the system determines exactly how much water each part of the field needs and when it is needed ^[120]^. The AI continuously learns from field data, allowing it to predict water requirements, spot unusual patterns, and automatically adjust watering schedules so plants get the right amount of hydration without being over or under watered ^[120]^. These AI powered irrigation systems lower energy use, support more resilient crops, and ultimately improve yields ^[123]^. Because they reduce unnecessary irrigation, they also promote environmental sustainability, an important benefit, as water scarcity is becoming a pressing global issue ^[124]^. Smart irrigation can also be paired with other digital farming tools, giving farmers remote access to their systems and real time alerts ^[123]^.

#### 3.4.3 AI in yield prediction and harvest optimization

Reliable yield forecasts are essential as they guide decisions about how to allocate resources, plan for market demand, and manage logistics, all of which directly influence both profitability and overall food security ^[125]^. In the past, estimating yields depended on historical trends or simple visual inspections, methods that overlook the constantly changing environmental conditions affecting crop growth ^[125]^. AI systems access and analyze data from a wide range of data sources such as satellite images, drone surveys, weather information, soil metrics, and previous yield records to reveal subtle patterns in plant development. This allows them to produce more accurate and timely yield projections throughout the growing season ^[125]^.

AI also plays an increasingly important role in optimizing harvest operations. Using AI generated yield maps along with real time sensor data, farmers can pinpoint the ideal moment to harvest, ensuring crops are collected when they are at peak quality ^[126]^. This precision helps reduce losses after harvest, improves product consistency, and boosts overall economic returns. For example, in fruit production, AI can monitor ripening stages and help determine the exact timing for picking ^[127]^. AI tools can also streamline the operation of harvesting equipment by planning efficient routes, saving fuel and labor costs, and optimizing machine use. When combined with technologies like autonomous harvesters, these systems make the entire harvesting process more efficient and sustainable ^[128]^.

### 3.5 AI enabled soil health and nutrient management

#### 3.5.1 Soil mapping and soil property prediction

Traditional approaches to soil mapping is slow, expensive, and limited in detail, which makes it difficult for farmers and land managers to apply precise, site specific strategies ^[129]^. AI powered systems in contrast, combine a wide range of datasets such as satellite and drone imagery, geophysical measurements, terrain characteristics, and past soil analyses to produce high-resolution soil maps ^[130]^. AI can estimate soil attributes, including organic carbon levels, pH, key nutrients like nitrogen, phosphorus, and potassium, as well as soil texture and moisture with high accuracy ^[131]^. Because they can detect subtle spatial patterns and relationships within complex datasets, they offer a far clearer and more dynamic picture of soil variability than archaic manual techniques ^[131]^. AI also supports predictive modeling, helping forecast how soil conditions may change in response to farming practices or environmental shifts ^[132]^. This gives farmers the ability to anticipate yield issues, refine nutrient management, and adopt more effective soil-conservation strategies. With AI enabled digital soil mapping, inputs such as fertilizer and irrigation can be applied exactly where they are needed, reducing waste and minimizing environmental impact; an important step toward higher productivity and sustainable farming 133, 134.

#### 3.5.2 AI for nutrient management and fertilization optimization

Instead of relying on fixed fertilization schedules, AI models examine a wide array of information such as soil test reports, plant tissue data, weather predictions, satellite imagery, and past crop performance to estimate nutrient needs at different stages of plant growth and under varying soil conditions, making it possible to design fertilization plans that adjust as the season progresses 135, 136. AI's ability to interpret complex relationships among soil properties, environmental influences, and crop requirements helps ensure nutrients are applied in the right amount, at the right moment, and exactly where they are needed ^[135]^. This approach improves nutrient-use efficiency, lowers fertilizer expenses, and reduces environmental risks. When paired with VRT, AI can guide equipment to deliver precise nutrient doses across different zones of a field ^[137]^. Sensors that monitor soil nutrient status and plant health in real time add another layer of precision, allowing farmers to quickly adjust fertilizer plans as conditions change ^[138]^.

#### 3.5.3 AI in soil microbiome analysis and bioremediation

The soil microbiome is made up of countless microorganisms gaining a clearer understanding of which is essential for improving soil health and building more sustainable agricultural systems ^[139]^. Because modern sequencing technologies generate enormous volumes of metagenomic, meta transcriptomic, and metaproteomic data, AI methods, especially machine learning and DL, have become invaluable for analyzing and interpreting these datasets ^[140]^. These models can pinpoint influential microbial species, predict their functions, and reveal the interactions that shape microbial communities insights that are difficult to obtain using traditional laboratory techniques alone ^[141]^. AI is also reshaping the field of bioremediation, offering new ways to treat contaminated soils. By examining variables such as pollutant type, soil conditions, and microbiome composition, In some cases, machine learning can even identify the best combinations of microorganisms or enzymes for breaking down specific contaminants, speeding up the design of tailored solutions ^[142]^. Real time monitoring powered by AI makes it possible to track the progress of remediation efforts and adjust treatment settings to improve efficiency and reduce costs ^[143]^. In addition, the computational demands of analyzing massive datasets can be substantial. Even so, ongoing advancements in AI and bioinformatics are steadily overcoming these obstacles ^[144]^. As these technologies mature, AI is expected to play an increasingly influential role in restoring damaged soils, improving fertility, and supporting more sustainable agricultural practices ^[144]^.

### 3.6 AI in climate resilient crop development

AI enabled platforms address limitations of current stress resistant crop development by combining high throughput sensors, robotics, and CV to gather extensive data on plant growth, development, and physiological responses under different stress scenarios ^[145]^. AI systems analyze these large datasets and detect meaningful patterns that reflect how plants cope with drought, heat, salinity, and other stresses ^[146]^. As such, AI identifies early signs of resilience before visible symptoms appear. These capabilities significantly speed up the discovery of valuable traits and selecting crop varieties with stronger adaptive potential. For example, AI-driven image analysis from drone footage or ground-based imaging can quantify key indicators such as leaf area, canopy temperature, and chlorophyll levels, all of which are essential for understanding stress responses ^[147]^. By modeling how different genotypes are likely to perform under future climate conditions, AI helps breeders prioritize the most resilient lines. It also improves our understanding of the complex relationships connecting genes, environment, and phenotype, offering a more complete picture of resilience mechanisms ^[148]^.

### 3.7 AI in livestock biotechnology

#### 3.7.1 AI for genetic improvement and breeding programs

Livestock breeding for the development of genetically improved animals has always been a manual, slow and labor intensive process relying heavily on observable traits and pedigree records ^[149]^.

AI models can estimate the genetic potential of individual animals with far greater accuracy than manual pedigree analysis ^[150]^. This includes traits such as growth performance, milk yield, fertility, and resistance to specific diseases information that helps breeders make more strategic and confident selection decisions ^[151]^. AI enhanced genomic selection predicts an animal's breeding value using genomic data rather than waiting for traits to appear later in life ^[152]^. This not only accelerates the selection process but also increases the overall rate of profitable yield ^[153]^. Machine learning tools can pinpoint the genetic markers underlying desirable traits, making it easier to guide breeding toward specific goals ^[150]^. AI can also help design mating plans that enhance beneficial traits while maintaining genetic diversity, reducing the risk of inbreeding and supporting long term sustainability in animal breeding ^[154]^.

#### 3.7.2 AI in livestock health and disease management

Conventionally animal health monitoring depends on visual inspection and manual observation, approaches that are time consuming, subjective, and often unable to catch early or subtle signs of illness ^[155]^. By overcoming these limitations by drawing on data from wearables, cameras, environmental monitors, and herd health records, AI is empowering sustainable farming ^[156]^. AI can identify potential health problems before any outward symptoms appear ^[157]^. This early warning allows farmers to isolate affected animals quickly, apply targeted treatments, and introduce preventive measures that help limit disease transmission and reduce reliance on expensive antibiotics ^[158]^. By analyzing data on feed intake, body condition, movement, and other indicators, AI can adjust feed composition and delivery in real time, helping prevent metabolic disorders and supporting optimal growth and productivity ^[159]^. On a larger scale, AI supports epidemiological surveillance by identifying risk factors and predicting potential disease outbreaks, allowing farmers and public health authorities to respond proactively ^[160]^. Integrating AI with genomic data provides insight into animals that may exhibit resistance to certain drugs or susceptibility to certain diseases. This information can guide selective breeding programs that strengthen herd health over the long term ^[159]^.

### 3.8 AI-driven biofertilizer and biopesticide development

#### 3.8.1 AI-driven biofertilizer development

AI is playing an increasingly important role in speeding up the development and refinement of biofertilizers; an essential, sustainable alternative to chemical fertilizers ^[161]^. Biofertilizers rely on living microorganisms that support plant nutrition by fixing nitrogen, solubilizing phosphorus, and producing compounds that stimulate plant growth ^[162]^. With the help of AI researchers can rapidly pinpoint microbial strains or microbial combinations with strong biofertilizer potential. AI contributes to several key stages of biofertilizer development. It can identify new growth-promoting rhizobacteria and other beneficial microbes from diverse ecosystems, revealing candidates that might otherwise be overlooked ^[163]^. By examining microbial genomes, AI can predict metabolic pathways involved in nutrient cycling or hormone production, making it possible to more strategically select or modify strains to enhance specific beneficial traits ^[164]^. AI also helps optimize fermentation and production processes, improving consistency, scalability, and cost-effectiveness. When combined with high-throughput screening technologies, AI enables rapid evaluation of microbial performance, significantly reducing the time required to bring new biofertilizers to market ^[165]^.

#### 3.8.2 AI-driven biopesticide development

Biopesticides, derived from natural materials such as animals, plants, bacteria, and certain minerals, are crucial for sustainable agriculture due to their targeted action, reduced environmental impact, and lower toxicity to non-target organisms ^[166]^.

AI-driven approaches facilitate biopesticide development by helping in identifying novel toxins, anti-feedants, or repellents from plant extracts or microbial metabolites ^[167]^. AI algorithms can analyze molecular structures to predict their biological activity and potential mechanisms of action, enabling targeted compound selection or modification to enhance desired properties ^[168]^. Furthermore, AI can optimize fermentation processes for large-scale production of microbial biopesticides, ensuring cost-effectiveness and consistency in product quality ^[169]^. The integration of AI with high-throughput screening platforms allows for rapid evaluation of biopesticide performance, significantly shortening the development cycle for new products ^[170]^.

**Fig. 5:**
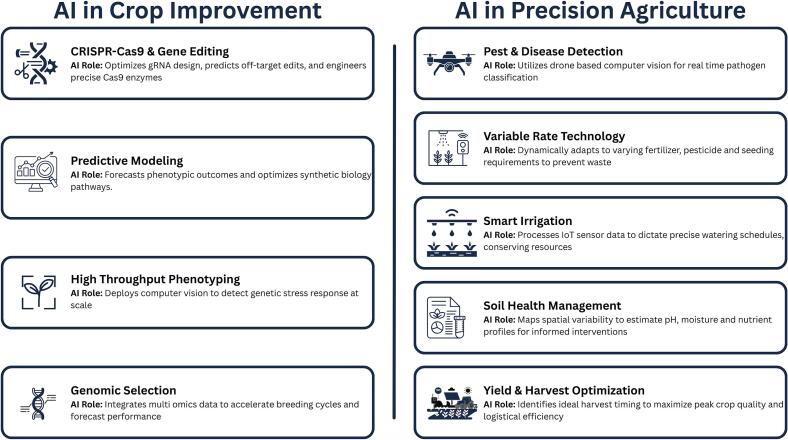
The role of AI in modern agriculture: A comprehensive illustration of the strategic applications of AI in crop improvement and field-level precision agriculture.

The deployment of AI in agricultural biotechnology spans from microscopic genetic manipulations to macroscopic field management. Fig. 5 provides an infographic summary of these integrations, delineating the specific role of AI in both crop improvement and precision agriculture. By replacing traditional workflows with data-driven solutions, these technologies collectively form the backbone of highly efficient farming systems.

### 3.9 Commercialization and research trends in agricultural biotechnology

**Fig. 6:**
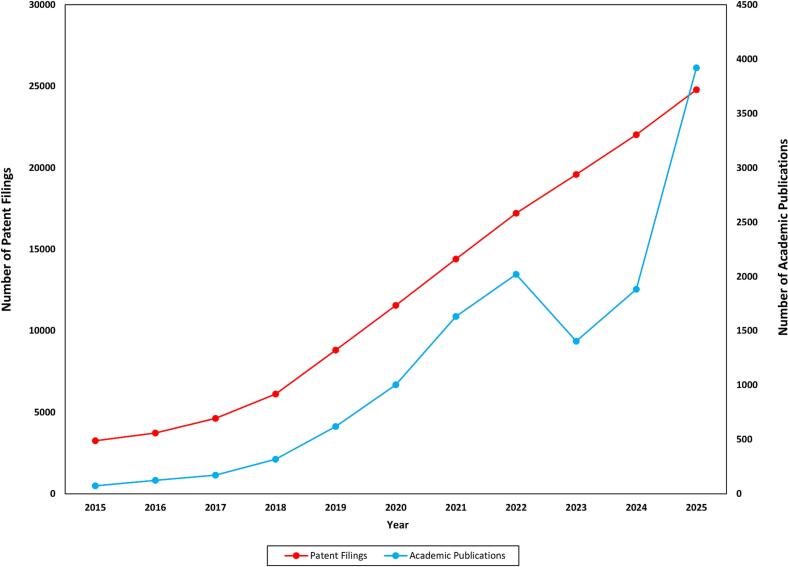
Dual Axis trend analysis (2015–2025) comparing the volume of AI driven agricultural academic publications versus global commercial patent filings. The exponential growth of patent filings, even during the academic contraction during 2023 confirms the industrial transition of these technologies.

The shift in agricultural AI is not only restricted within academia; it is being aggressively mirrored by commercial intellectual property generation and private equity interests. An analysis of global patent data reveals an exponential commercialization trajectory. Between January 2015 and December 2025, the volume of AI-powered agricultural patents and patent applications skyrocketed (Fig. 6). Using the string: (“artificial intelligence” OR “machine learning” OR “deep learning” OR “computer vision” OR “neural network”) AND (“agriculture” OR “biotechnology” OR “crop” OR “farming” OR “harvest”) we performed a systematic search for patent filings in Lens (https://www.lens.org/). We only included “Granted Patents” and “Patent Applications” to better reflect the commercial landscape and industry interest in the field. Date range was set from 1 January 2015 to 31 December 2025. To gain insight into academic interests, we utilized the data gathered in section 2.1.3.

While academic outputs (as scholarly publications) and commercial intellectual property (as patent filings) followed a similar trajectory, academic outputs experienced a notable contraction in 2023, likely due to post-COVID publishing delays or database indexing lags. Commercial patent filings, however, were completely immune to this fluctuation. This industrial growth is supported by aggressive capital allocations. According to leading Agri-tech investment reports, funding for consumer-focused downstream (consumer focused) technologies reached 6.1 billion USD, representing a 38% year over year increase driven primarily by large deals in the United States and India. Additionally, midstream technology funding increased at around 41% year over year, securing 1.7 billion USD in total investments 171, 172.

This insight confirms that the transition from archaic agronomy to AI powered architectures is a globally funded, commercially viable paradigm shift, rapidly transitioning from prototypes to scalable, market-ready solutions.

### 3.10 Challenges and ethical considerations in AI based agricultural biotechnology

#### 3.10.1 The digital divide and socioeconomic implications

One of the most pressing ethical concern in agricultural AI is the widening “digital divide” ^[173]^. The infrastructure required for AI, such as robust internet infrastructure, expensive IoT networks, and high-performance computing is predominantly accessible to farmers located in high-income nations. Consequently, smallholder farmers, particularly in developing nations such as South Asia and Sub-Saharan Africa are severely disadvantaged due to lack of access to such resources ^[174]^. Without targeted policy interventions and affordable “Small AI” solutions designed to operate on minimal bandwidth, the proliferation of AI threatens to render smallholder operations economically unsustainable, concentrating agricultural power in the hands of rich farmers and corporations ^[175]^. Small AI solutions are typically developed using computational optimization techniques such as model quantization, knowledge distillation, and lightweight neural network architectures. These approaches reduce model size and computational requirements while preserving acceptable predictive performance. In addition, edge-computing-based deployment allows AI models to process data locally on mobile devices or low-cost agricultural sensors, minimizing dependence on continuous internet connectivity and high-bandwidth infrastructure. Such approaches can make AI technologies more accessible to smallholder farmers in resource-constrained regions 176, 177, 178.

#### 3.10.2 Data ownership, privacy, and corporate monopoly

Predictive agricultural models are heavily reliant on massive datasets detailing soil health, crop genetics, and historical yields ^[179]^. This raises a critical concern of data sovereignty: who truly owns the data generated by a farmer's field? ^[180]^. Agri-tech giants often harvest this field level data to train proprietary algorithms, creating a massive power asymmetry 180, 181, 182, 183. Farmers often lack the legal assistance to protect their proprietary agricultural data from being exploited by large corporations. Establishing secure, federated data sharing models and clear data governance policies is essential in ensuring that farmers retain ownership and are fairly compensated for the intelligence their land generates ^[184]^.

#### 3.10.3 Algorithmic bias

Historically, many foundational agricultural models have been trained on datasets derived from western, temperate climates focusing on major commodity crops such as wheat ^[185]^. When these models are deployed in entirely different agro-ecological zones such as tropical regions for cultivating specialized rice varieties or native crops, they can suffer from severe algorithmic bias which can lead to erroneous decision making, such as incorrect irrigation or fertilization recommendations, leading to potential devastating outcomes ^[186]^.

#### 3.10.4 Unintended ecological consequences

General purpose ML systems in agriculture are typically optimized for a singular metric: maximizing short-term yield. In natural environments, following an AI's aggressive fertilization or pesticide recommendations to boost immediate output may disregard concerns regarding long term soil microbiome, water toxicity and local biodiversity ^[185]^. Therefore, the ethical deployment of AI must involve “ethics by design” ensuring that AI systems account for holistic, long term ecological sustainability rather than just immediate economic returns ^[184]^.

### 3.11 Future prospects of AI in agricultural biotechnology

#### 3.11.1 Autonomous farms

Unlike traditional AI which require explicit human intervention to execute tasks agentic AI acts as autonomous decision makers capable of long term planning and execution of tasks ^[187]^. In future, Agentic AI powered farms will coordinate multiple sub-agents such as smart irrigation networks and robotic weeders and harvesters. For example, if an AI agent detects a pest outbreak via camera feed or data from integrated sensors, it will automatically apply a targeted pesticide in the affected region of the farm ^[188]^.

#### 3.11.2 Quantum computing in agriculture

As modern computers are reaching the limits of their processing power, quantum computing is emerging as the next frontier in Agriculture Quantum computers can process vast multivariable data faster than currently available computing resources ^[189]^. Recently researchers demonstrated that combining quantum computing with neural networks allows for the precise simulation of soil nutrient pathways and symbiotic root-microbe relationships. This optimizes biofertilizer formulations, resulting in significantly higher crop yields with reduced chemical runoff ^[190]^.

## 4 Summary

The deployment of AI in agricultural biotechnology represents a paradigm shift empowered by modern advanced technological tools. Advances in computational capabilities have expanded the capacities of AI technologies, empowering them not only to enhance existing agricultural practices but also to facilitate the development of entirely new approaches to long standing challenges. AI is addressing the increasing need for sustainable and efficient food production. AI powered solutions are optimizing crop breeding, enhancing pest control, improving resource utilization, ultimately enabling precision farming. These advancements are driving the development of more resilient and productive agricultural systems capable of higher productivity than traditional approaches.

Nevertheless, the widespread adoption of AI and the commercialization of AI enabled technologies, as evidenced by surging patent volumes also raises several important ethical and social concerns, particularly regarding the nature and use of the data required to train and operate these systems. Issues including algorithmic bias, digital divide, data privacy and ownership and the limited interpretability of complex neural networks, often known as the “black box” problem, must be address carefully. Addressing these challenges will require the establishment of robust regulatory frameworks, effective oversights and the development of explainable AI that improve transparency and accountability.

The future of AI is expected to be shaped by continuous technological innovation. Emerging developments such as AG, Agentic AI, autonomous farms and advances in quantum computing hold the potential to further accelerate discovery and innovation with agricultural biotechnology and related fields. As AI systems increasingly function as collaborative tools in expanding the frontiers of scientific knowledge, their deployment will need continuous empirical evaluation, thoughtful governance and ethical oversight to ensure that their capabilities are harnessed responsibly for the broader benefit of society.

## Ethics, consent to participate, and consent to publish declarations

Not applicable, as no human or animal subjects were involved in this study.

## CRediT authorship contribution statement

**Zubaer Hossen:** Writing – review & editing, Writing – original draft, Conceptualization. **Md. Naim Uddin Forhad:** Writing – review & editing, Writing – original draft, Conceptualization. **Md. Rifat Bin Ayez:** Writing – review & editing, Writing – original draft, Conceptualization. **Shusmita Karmaker:** Writing – review & editing. **Md Nur Islam:** Writing – review & editing. **Md. Sarowar Hossain:** Writing – review & editing. **Md. Enamul Haque:** Writing – review & editing. **Md. Nazmul Hasan:** Writing – review & editing. **Md Mahmudul Islam:** Writing – review & editing, Writing – original draft, Supervision, Conceptualization.

## Clinical trial number

Not applicable.

Funding

This research did not receive any specific grant.

## Declaration of competing interest

The authors declare that they have no known competing financial interests or personal relationships that could have appeared to influence the work reported in this paper.

## Data Availability

Data included in article/supp. Material.
